# Effects of liquid surface tension on gas capillaries and capillary forces at superamphiphobic surfaces

**DOI:** 10.1038/s41598-023-33875-9

**Published:** 2023-04-26

**Authors:** Mimmi Eriksson, Per M. Claesson, Mikael Järn, Viveca Wallqvist, Mikko Tuominen, Michael Kappl, Hannu Teisala, Doris Vollmer, Joachim Schoelkopf, Patrick A. C. Gane, Jyrki M. Mäkelä, Agne Swerin

**Affiliations:** 1 RISE Research Institutes of Sweden, 11486 Stockholm, Sweden; 2 Division of Surface and Corrosion Science, Department of Chemistry, School of Engineering Sciences in Chemistry, Biotechnology and Health, KTH Royal Institute of Technology, 10044 Stockholm, Sweden; 3 Department of Physics at Interfaces, Max Planck Institute for Polymer Research, 55128 Mainz, Germany; 4 Omya International AG, 4665 Oftringen, Switzerland; 5 Department of Bioproducts and Biosystems, School of Chemical Engineering, Aalto University, 00076 Aalto, Finland; 6 Faculty of Technology and Metallurgy, University of Belgrade, Karnegijeva 4, 11000 Belgrade, Serbia; 7 Physics Unit, Aerosol Physics Laboratory, Tampere University, 33014 Tampere, Finland; 8 Department of Engineering and Chemical Sciences, Karlstad University, 65188 Karlstad, Sweden

**Keywords:** Chemical physics, Thermodynamics

## Abstract

The formation of a bridging gas capillary between superhydrophobic surfaces in water gives rise to strongly attractive interactions ranging up to several micrometers on separation. However, most liquids used in materials research are oil-based or contain surfactants. Superamphiphobic surfaces repel both water and low-surface-tension liquids. To control the interactions between a superamphiphobic surface and a particle, it needs to be resolved whether and how gas capillaries form in non-polar and low-surface-tension liquids. Such insight will aid advanced functional materials development. Here, we combine laser scanning confocal imaging and colloidal probe atomic force microscopy to elucidate the interaction between a superamphiphobic surface and a hydrophobic microparticle in three liquids with different surface tensions: water (73 mN m^−1^), ethylene glycol (48 mN m^−1^) and hexadecane (27 mN m^−1^). We show that bridging gas capillaries are formed in all three liquids. Force-distance curves between the superamphiphobic surface and the particle reveal strong attractive interactions, where the range and magnitude decrease with liquid surface tension. Comparison of free energy calculations based on the capillary menisci shapes and the force measurements suggest that under our dynamic measurements the gas pressure in the capillary is slightly below ambient.

## Introduction

Gas capillary formation in liquids and the corresponding issue of capillary condensation of liquids has a broad and general interest in any system where solids, liquids and gases are present simultaneously. Capillary forces become of increasing importance as the dimension of the objects are reduced as in nanotechnology^1^ where it for instance is of paramount importance for adhesion and friction forces^2–4^. It is of equal importance in established technologies such as flotation^5^, used for mineral recovery and in recycling processes. The presence of dissolved gas influences hydrophobic interactions in general, even though all that is referred to as hydrophobic interactions is not caused by capillary evaporation^6–9^. Nevertheless, the two subjects are certainly related. Superhydrophobic and superamphiphobic surfaces are of great current interest as such surfaces have been shown to, for instance, reduce ice adhesion^10^ and have important applications in biomaterial design^11^, modified wood^12^ and corrosion protection^13^. Many other applications have also recently been discussed^14^. A prerequisite for development of this broad range of applications is to further the understanding of events occurring at solid–liquid–gas interfaces, which also constitutes the aim of the research presented in this article.

A superamphiphobic surface repels both high surface tension water and other polar or non-polar low surface tension liquids^15,16^. Liquid drops on a superamphiphobic surface adopt an almost spherical shape and display a high contact angle (typically > 150°). In addition, the low adhesion to the surface causes the drops to roll off easily at low tilt angles (typically < 5–10°). Superamphiphobicity is generally reached if allied with micro/nanoscale surface features and is most often achieved using structures with high re-entrant curvature and a low surface energy^17,18^. The required surface structure has been achieved by e.g. well-defined mushroom-like shapes^19,20^, or random sub micrometer structures with overhang geometry such as coatings of nanofilaments^21,22^ or nanoparticles^23–25^. The thermodynamics of different wetting states and the transition between these have been reported in detail^26^. With the appropriate combination of surface structure and chemistry, a liquid drop will be in the so-called Cassie-Baxter wetting state^27^. Here, the liquid is suspended on top of the surface structures with a gaseous layer trapped in the void structure underneath, and in this way the solid–liquid contact area is reduced.

Traditional wettability characterization by contact angle goniometry has been suggested to involve uncertainties for surfaces displaying very high contact angles^28,29^. As an alternative or complement, force-based methods can be utilized^30^. These have the advantage of being very sensitive and offer the possibility to capture microscale variations in wetting properties^31^. Force measurements also contribute to increased general understanding of super liquid-repellence and the governing mechanisms. Measurements of interactions involving at least one liquid-repellent surface across a non-wetting liquid have so far mostly been limited to superhydrophobic surfaces across water. To the best of our knowledge there are no reports of force measurements utilizing a superamphiphobic surface in low-surface-tension liquids, which are highly relevant in materials science research.

It is known that forces between a superhydrophobic surface and a (super)hydrophobic particle are strongly attractive with interactions ranging up to several micrometers on separation^32–36^. These attractive forces are due to the formation of a bridging gas (air or vapor) capillary between the two surfaces^37,38^. During separation, the gas capillary has been observed to increase in size as gas present in the surface layer can act as a reservoir allowing the capillary volume to increase^34^. How the liquid surface tension affects the interactions between superhydrophobic surfaces has been studied by adding surfactants^35^ or ethanol^33^ to water. Force-distance curves in agreement with gas capillary formation and growth during separation where observed for liquid surface tensions down to 40 mN m^−1^ (20 vol% ethanol)^33^. However, the likelihood of observing such forces decreased as compared to that in pure water. It is unclear whether a gas capillary is formed also for low-surface-tension liquids and how the surface tension affects the range and magnitude of the force, as well as the size and evolution of the gas capillary. It is clear that a reduction in surface tension will reduce the free energy penalty of creating the gas–liquid interface of the capillary and based on only this fact one could conclude that capillaries would form more easily in a low surface tension liquid. However, the driving force for forming the capillary is the replacement of solid–liquid contacts with solid–gas contacts, and this free energy reduction is likely less for low surface tension liquids. Thus, experiments are needed in order to clarify the effect of surface tension on capillary formation at (super)hydrophobic surfaces. A further aspect is that gas solubility is expected to increase with decreasing liquid surface tension^39^ which highlights the importance of further understanding of superamphiphobicity.

In this study, we used colloidal probe atomic force microscopy (AFM) with an extended piezo-range to measure interactions between a superamphiphobic surface and a hydrophobic microparticle in three liquids with different surface tensions: water (73 mN m^−1^), ethylene glycol (48 mN m^−1^) and hexadecane (27 mN m^−1^). Laser scanning confocal microscopy was utilized for visualizing the events occurring during force measurements. The formation of capillary bridges and its evolution was monitored, and from the capillary size and meniscus shapes the attractive forces were calculated.

We show for the first time that gas capillaries form at superamphiphobic surfaces in low-surface-tension and non-polar liquids, showing the mechanism to be physically similar to that of hydrophobic surfaces interacting in water.

## Results and discussion

### Structure and chemical composition of the superamphiphobic surface

Nanostructured TiO_2_/SiO_2_ coatings were prepared on thin cover glasses by liquid flame spray (LFS)^24^. A liquid solution containing organometallic precursor molecules is atomized in a hydrogen–oxygen high-temperature flame (> 2500 °C)^40^. The solution evaporates in the flame and the organometallic molecules react to form nanoparticles which collect on the surface^12,24,41^.

**Figure 1:**
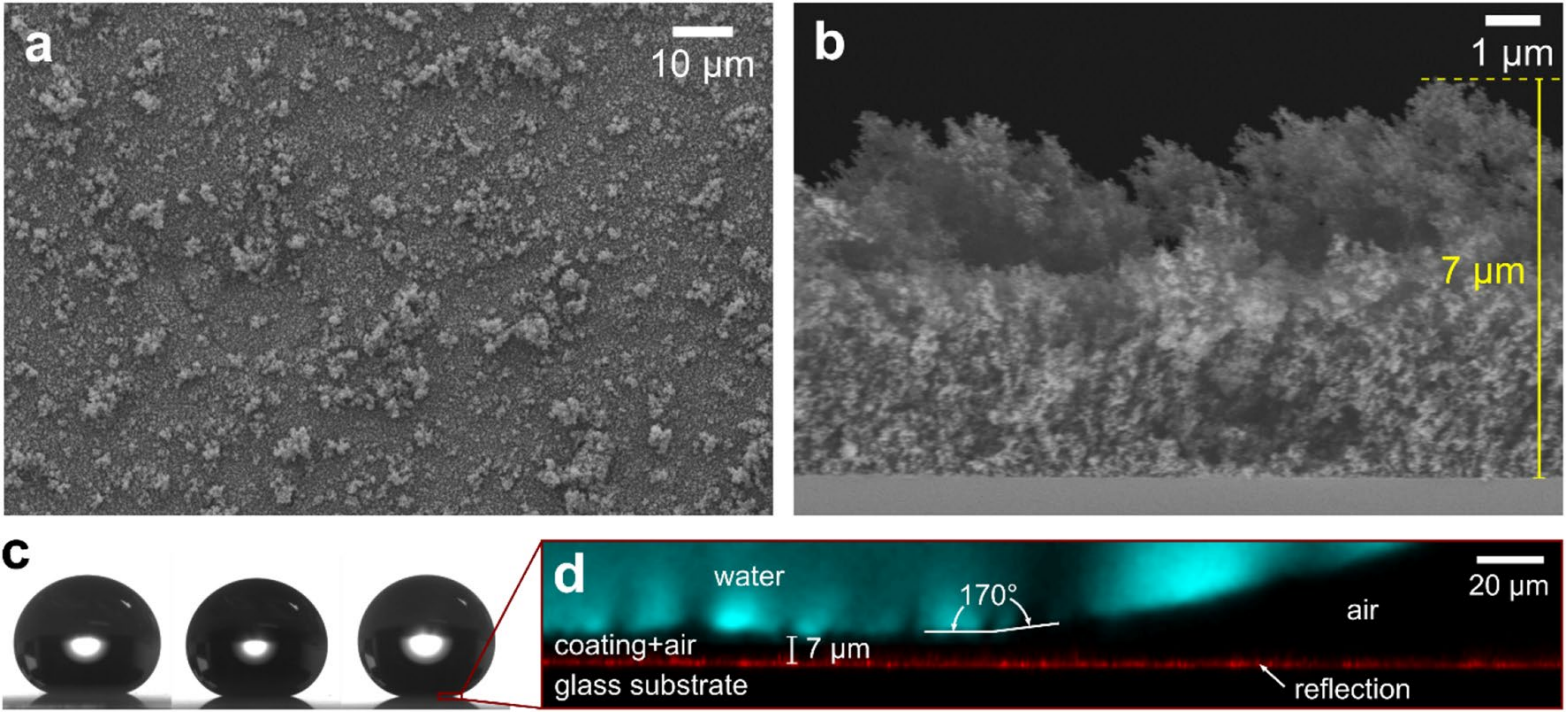
(**a**) Top-view and (**b**) cross-sectional SEM images of a superamphiphobic coating, (**c**) shape of 6 μL drops of (from left to right) hexadecane, ethylene glycol and water resting on the surface, and (**d**) laser scanning confocal microscopy image of a water drop labeled with fluorescent dye (1 mg L^−1^).

Scanning electron microscopy (SEM) images reveal a highly porous coating with hierarchical roughness (Fig. 1a). Cross-sectional images show a coating thickness around 4 μm at the thinnest locations with the highest protrusions reaching approximately 7 μm (Fig. 1b). The TiO_2_/SiO_2_ coatings were additionally coated with a thin silica shell (to prevent photocatalytic degradation of the silane) and further surface modified with 1H,1H,2H,2H-perfluorooctyl-trichlorosilane in order to achieve superamphiphobicity.

Chemical analysis of the superamphiphobic surface was performed by X-ray photoelectron spectroscopy (XPS) showing the presence of Si, Ti, O, C and F in the top 2–10 nm surface layer (Fig. S1a, Supporting Information). The high-resolution spectrum in the carbon C1s region (Fig. S1b, Supporting Information) gives a C/F ratio of 0.5 and a CF_3_/CF_2_ ratio of 0.25, which is close to ratios calculated from the chemical structure of the fluorosilane (C/F = 0.6, CF_3_/CF_2_ = 0.2). Since XPS is very surface sensitive, we expect most of the signal from the fluorosilane and silica layers and little information from the bulk coating layer of TiO_2_/SiO_2_. The presence of a small Ti peak suggests the fluorosilane and silica layers being only one or a few nanometers thick, or incomplete in some areas. The chemical composition of the underlying nanostructured TiO_2_/SiO_2_ coating layer, prepared in the same way as our surfaces, was studied by Teisala et al*.*^24^, where energy dispersive X-ray spectroscopy (EDS/EDX) showed a Ti/Si ratio of 97.9/2.1 wt%/wt%.

### Contact angles from goniometry and confocal images

After surface modification, the coated samples were superamphiphobic as drops of water, ethylene glycol and hexadecane were seen to take an almost spherical shape (Fig. 1c), each displaying very high contact angles (Table 1). It is worth noting that for such high contact angles (≳ 150°) the gap between the solid and liquid close to the contact line is too small to be accurately determined, leading to large observational errors when using optical goniometry. With confocal microscopy we observe that the contact angles are in fact around 170° (Fig. 1d). A Cassie-Baxter-type wetting state is visualized in the confocal image with the gaseous layer underneath a droplet being approximately 7 ± 1 μm thick for all three liquids (Fig. S2, Supporting Information). The thickness of the gaseous layer corresponds to the height of the highest protrusions of the coating (Fig. 1b). This suggests that the liquid is largely suspended on top of these protrusions. The (lateral) adhesion to the surface by all three liquids was found to be very low as revealed by roll-off (tilt) angles ≤ 2° for 10 μL droplets (Table 1). For water, the roll-off angle was too low to be measured, as droplets either escaped as soon as the droplet application needle was detached (the sample being horizontally levelled without any apparent tilt) or rolled off as soon as the tilting started.

**Table 1:** Average values with standard deviations of the apparent advancing (*θ*_a_) and receding (*θ*_r_) contact angles as well as the roll-off angles (*RA*) for 10 μL drops of water, ethylene glycol and hexadecane on the superamphiphobic surface, measured with goniometry.

Liquid	*θ*_a_ (°)	*θ*_r_ (°)	*RA* (°)
Water	161 ± 1	159 ± 1	< 1
Ethylene glycol	161 ± 1	151 ± 2	2 ± 1
Hexadecane	160 ± 2	150 ± 5	2 ± 1

### Surface forces and observation of gas capillaries

Spherical glass particles (radius *R* = 14–16 μm) were attached to tipless cantilevers (spring constants *k*_z_ = 12–19 N m^−1^) and surface modified with 1H,1H,2H,2H-perfluorooctyl-trichlorosilane. The respective force between the particle and the superamphiphobic surface in water, ethylene glycol and hexadecane was measured using colloidal probe AFM. An inverted confocal microscope was used for imaging during force measurements. The liquid phases were visualized by adding fluorescent dyes (Fig. S3, Supporting Information) at low concentrations (10–50 mg L^−1^), and the fluorescence from the dyed liquids and light reflected from the interfaces were detected simultaneously to obtain the size and shape of the gas capillary (Fig. S4, Supporting Information).

Light reflected from the liquid–gas and substrate-gas interfaces at the superamphiphobic surface demonstrates the presence of a gaseous layer originating from the non-wetted regions within the porous coating underneath each of the liquids. The gaseous layer is approximately 4 μm thick for all three liquids. This thickness is in the same order as the thinnest part of the coating layer (determined from cross-sectional SEM images). However, as discussed above, for a liquid droplet resting on the surface, the gaseous layer is thicker. The observed lower thickness in force experiments as compared to the situation of a liquid droplet could have several reasons. For instance, the liquid might partially penetrate the coating layer due to partial dissolution of gas into the liquid. Further, in the process of trapping the liquid between the surfaces in the AFM, the liquid is pressed against the superamphiphobic surface which could cause the observed lower thickness of the gaseous layer.

**Figure 2:**
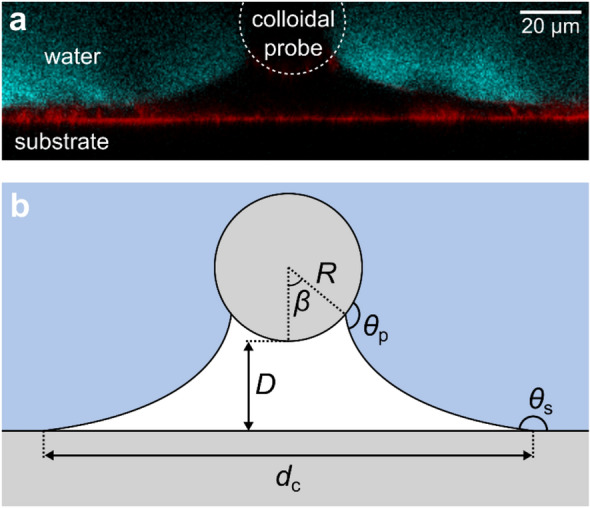
(**a**) Laser scanning confocal image of a gas capillary between the particle and superamphiphobic surface captured during retraction in water and (**b**) illustration of a catenary shape gas capillary between a spherical particle (radius *R*) and a flat surface at separation *D*, with the diameter of the de-wetted area on the flat surface (*d*_c_), the angle defining the de-wetted area on the particle (*β*) and the microscopic contact angles at the gas–liquid interface of the flat surface (*θ*_s_) and particle (*θ*_p_).

**Figure 3:**
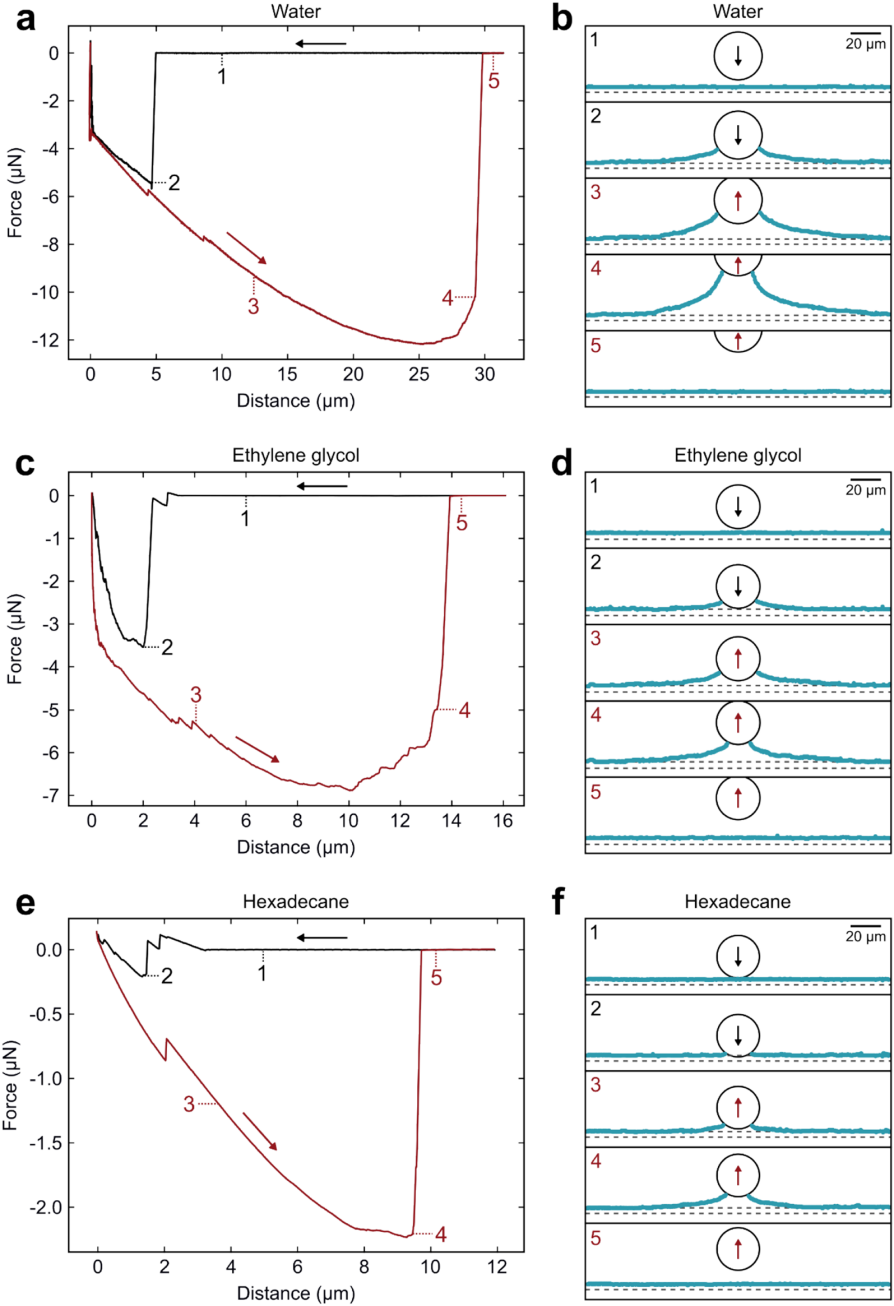
Representative force-distance curves (approach in black, retraction in red) with corresponding menisci shapes at the different positions (1–5) for measurements in (**a**,**b**) water, (**c**,**d**) ethylene glycol and (**e**,**f**) hexadecane. In (**b**), (**d**) and (**f**), the shape of the liquid–gas interface is plotted in cyan, the positions of the liquid–gas and glass-gas reflections as dashed lines and the particle position as a black circle. The piezo expansion rate was 0.2 µm s^−1^.

Confocal imaging during force measurements, when the particle is moving towards and away from the superamphiphobic surface, revealed a bridging gas capillary with a catenary shape (Fig. 2) between the particle and the superamphiphobic surface in all three liquids (Videos S1, S2 and S3, Supporting Information). From typical force-distance curves recorded in the three liquids we observe qualitative and quantitative differences (Fig. 3 and Table 2). A valid concern is local heating of the liquid by the low intensity laser used in the measurements. A local heating would give rise to liquid flow that could facilitate formation of gas capillaries on approach and promote breakage of the capillaries during separation of the surfaces. The heating of a medium by laser depends on the absorption of the medium to the laser irradiation wavelength (473 nm). For all three liquids the absorption at this wavelength is low, suggesting limited local heating in all cases. Should some heating take place through radiation absorption by surfaces, the temperature rise will depend on the liquid heat capacity, which for water, ethylene glycol and hexadecane are 4.19, 2.42 and 2.21 J/gK, respectively. Thus, for a given energy absorbed the largest temperature gradient is expected for hexadecane. If local heating would be of significant importance, one would thus expect gas capillaries to form most easily on approach in hexadecane and the capillary to rupture most easily on separation in this liquid. In contrast, the experiments show that gas capillaries are most difficult to form on approach in hexadecane even though they also break most easily in this liquid. Further, the heat capacities of hexadecane and ethylene glycol are similar, but the capillary formation and evolution process in these two liquids are significantly different. Thus, we conclude that local heating is not a prime reason for the differences observed in the three liquids as reported below.

**Table 2:** Average values with standard deviations of the range of attraction on approach (*D*_a_) and retraction (*D*_r_), the maximum attractive force encountered on approach (*F*_a_) and retraction (*F*_r_) obtained from force measurements in water, ethylene glycol and hexadecane.

Liquid	*D*_a_ (μm)	*D*_r_ (μm)	*F*_a_ (μN)	*F*_r_ (μN)
Water	4.8 ± 0.7	29.4 ± 1.2	5.5 ± 0.6	12.1 ± 0.6
Ethylene glycol	2.7 ± 1.3	13.3 ± 1.1	3.3 ± 0.9	7.0 ± 0.1
Hexadecane	1.5 ± 0.3	9.3 ± 1.0	0.12 ± 0.05	2.0 ± 0.1

#### Forces measured on approach

During a force measurement, the particle is first approaching the superamphiphobic surface (Fig. 3, black lines). When the separation is large, the force is zero (Fig. 3, point 1). At some point when the separation becomes sufficiently small an attractive force (defined as negative) appears as a gas capillary is formed between the particle and the surface (Fig. 3, point 2). The distance corresponding to the onset of this attractive force will be referred to as the range of attraction observed on approach, *D*_a_. In water, *D*_a_ is around 5 ± 1 μm (Table 2), and the attraction is very strong and appears suddenly (Fig. 3a). Corresponding confocal images at *D*_a_ show the formation of a large gas capillary forming a bridge surrounded by the liquid phase (Fig. 3b). The large capillary gives rise to the attractive force observed on approach. The maximum attractive force on approach *F*_a_ typically has a value of 5.5 ± 1 μN and is reached just below *D*_a_ for measurements in water.

In ethylene glycol, a small step in the attractive force is observed after *D*_a_ and before *F*_a_ is reached (Fig. 3c). This step corresponds to the formation of a small gas capillary at the first attractive step (Fig. S5, Supporting Information). When the particle continues to approach the surface, a larger gas capillary is formed giving rise to a stronger attractive force *F*_a_. A step in the attractive force upon formation of an initial small gas capillary on approach is typically also observed in hexadecane (Fig. 3e). The stepwise meniscus formation is due to pinning of the three-phase contact line (TPCL), which occurs more frequently the lower the surface tension of the liquid.

**Figure 4:**
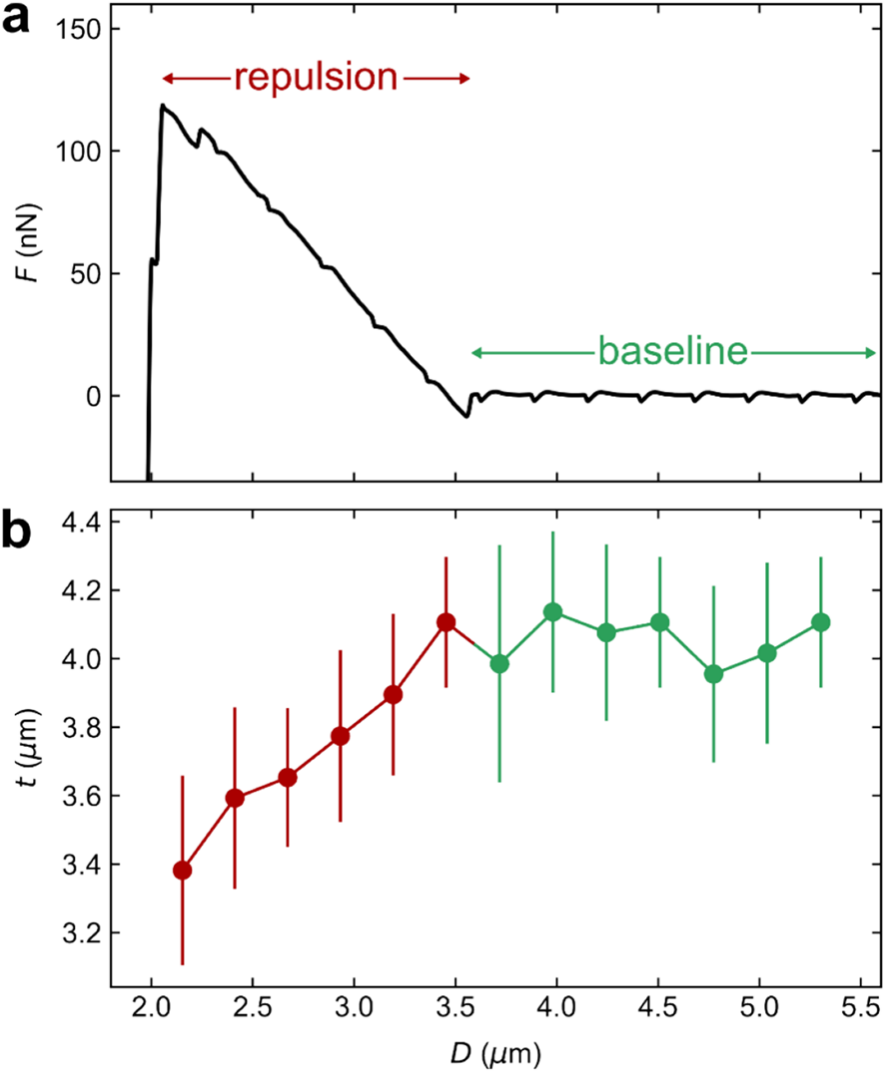
Example of a measurement in hexadecane where a measured repulsive force (*F*) is observed on approach with the corresponding gaseous layer thicknesses (*t*): (**a**) *F* as a function of separation *D* zoomed-in on the repulsive part, (**b**) *t* observed directly underneath the particle at the corresponding separation. The error bars show the standard deviation at each point. The piezo expansion rate was 0.2 µm s^−1^.

In addition, in hexadecane a small repulsion (≈ 0.12 μN) is typically observed prior *D*_a_. A small repulsive force is in some cases also observed in ethylene glycol but never in water (Fig. S6, Supporting Information). This repulsion appears *before* the capillary has formed and is thus not due to a fully repulsive capillary force as observed in some cases^42,43^. Instead it is due to a repulsive force between the colloidal probe and the air/liquid interface, the air being trapped within the surface structuration. Due to the low dielectric constant of hexadecane it is not likely to be a double-layer force, as has been reported for bubbles interacting with hydrophilic silica surfaces^44^. As the refractive index of the liquid is higher than that of air and fluorocarbon it is also not due to a repulsive van der Waals force. Instead we suggest that it is due to a *short-range* repulsion related to removing adsorbed liquid molecules from the colloidal particle, most similar to solvation forces observed between two solid surfaces. Such a force is only expected when the liquid contact angle is below 90°, rationalizing why it is absent in water. The action of this short-range repulsive force results in significant deformation of the gas–liquid interface at the superamphiphobic surface, and this compression of the gaseous layer can be seen in the confocal images (Fig. 4) (see Video S3, Supporting Information, at 61–63 s). Quantitatively, the gaseous layer thickness decreased from 4.1 ± 0.3 μm (Fig. 4b, green symbols) to 3.4 ± 0.3 μm (Fig. 4b, red symbols) before the gas capillary is formed. A deformation of the gas–liquid interface is supported by the linear increase of the deformation (decrease in distance) with increasing force, as the response of a flat interface to deformation leads to an almost linear response for small deformations with low curvature^45^. Further, the energy required for a certain deformation of the gas–liquid interface should scale with the liquid–gas surface tension, and indeed the ratio of the slopes of the repulsive part in ethylene glycol and hexadecane (≈ 1.7–1.8) is close to the ratio of their surface tensions (1.8).

#### Forces measured on retraction

After the particle contacts the superamphiphobic surface at zero separation, the cantilever is retracted (Fig. 3, red lines). An attractive force is again observed as the distance is increasing during retraction. The bridging gas capillary is intact and spreads on the superamphiphobic surface as the attractive force increases (Fig. 3, point 3). In water, the attractive force continues to increase until a separation of up to 25 μm at the maximum force *F*_r_ on retraction, which typically has a value of *F*_r_ ≈ 12 ± 1 μN (Table 2).

In ethylene glycol and hexadecane, the largest attractive forces observed on retraction are smaller, *F*_r_ ≈ 7 μN and *F*_r_ ≈ 2 μN, respectively, and the force minimum is reached at smaller separations than in water. The smaller values correspond to smaller gas capillaries (Fig. 3d,f). After *F*_r_, the attractive force decreases, and the capillary ruptures at the separation distance *D*_r_ (Fig. 3, point 4) and the force returns to zero (Fig. 3, point 5).

In water, the capillary typically persists up to a separation of 30 μm, whereas in ethylene glycol and hexadecane the range is smaller with typical values of *D*_r_ = 13.3 μm and *D*_r_ = 9.3 μm, respectively (Table 2). Steps in the force curve are often observed in the retract part of the force-distance curves in all three liquids (*e.g.* in Fig. 3a at *D* ≈ 5 μm, Fig. 3c at *D* ≈ 4 μm and Fig. 3e at *D* ≈ 2 μm). In many cases, the corresponding confocal images before and after a step in the attractive force show depinning of the TPCL on the superamphiphobic surface (Fig. S7, Supporting Information). In cases where this distinct jump of the TPCL is not observed in the confocal image, it is likely that it does not occur in the plane of the 2D cross-section of the image, or it may be too small to resolve from the confocal images. The force and distance where these depinning events occur depends on the position on the surface, which is a consequence of the heterogeneous nature of the superamphiphobic coating. The variation of many of the key features discussed above is reported in Table 2.

#### Characterization of the gas capillary evolution

**Figure 5:**
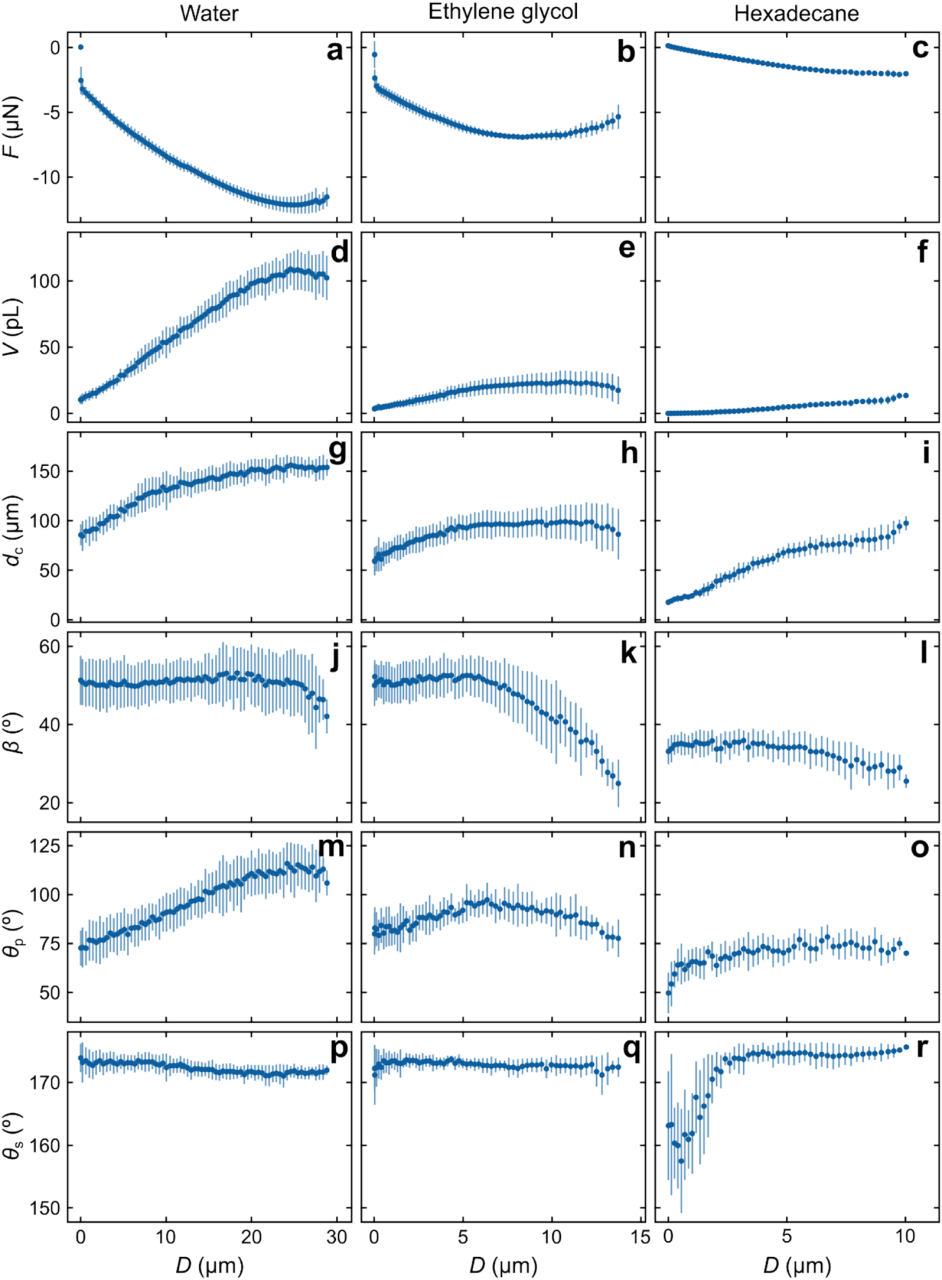
Diagrams of (**a**–**c**) force *F*, (**d**–**f**) capillary volume *V*, (**g**–**i**) diameter of the de-wetted area on the superamphiphobic surface *d*_c_, (**j**–**l**) angle *β* defining the de-wetted area on the particle and microscopic contact angles at the gas–liquid interface of the particle *θ*_p_ (**m**–**o**), and on the superamphiphobic surface *θ*_s_ (**p**–**r**) as a function of separation *D* for measurements in water, ethylene glycol and hexadecane. Error bars show the standard deviations. The piezo expansion rate was 0.2 µm s^−1^.

From the shape of the capillary meniscus under dynamic force measurement obtained from analysis of the confocal images (Supporting Information), we determined the capillary volume *V*, the diameter of the de-wetted area on the flat surface *d*_c_, the angle defining the de-wetted area on the particle *β*, and the dynamic microscopic contact angles at the gas–liquid interface on the superamphiphobic surface *θ*_s_ and particle *θ*_p_, respectively (Fig. 2b). Initially, the gas capillaries grow in volume during separation in all three liquids (Fig. 5d–f). In water, the capillary typically reaches maximum volumes larger than 100 pL (*V*_max_ = 118 ± 13 pL), while in ethylene glycol and hexadecane the maximum observed volumes are much smaller (*V*_max_ = 25 ± 6 pL and *V*_max_ = 9 ± 3 pL, respectively). The volume growth is expected to be mainly due to inflow of gas from the gaseous layer at the superamphiphobic surface but may also be partly due to dissolved gases in the liquid diffusing into the capillary. However, we note that the air solubility is highest in hexadecane where capillaries are smallest and lowest in water where capillaries are largest (Supporting Information), which suggests that gas diffusion from the liquid is less important than diffusion from the trapped air layer on the superamphiphobic surface.

Due to a low adhesion to the superamphiphobic surface of all three liquids, we observe a low degree of pinning to the surface and the TPCL is moving over the superamphiphobic surface during the main part of the retraction, with the exception of pinning events leading to small steps in the force curve (Fig. 5g–i). The capillary width on the superamphiphobic surface increases and reaches maximum values of *d*_c_ = 166 ± 9 μm, *d*_c_ = 104 ± 14 μm and *d*_c_ = 83 ± 10 μm in water, ethylene glycol and hexadecane, respectively. In contrast, the TPCL on the particle is typically pinned as long as the attractive force continues to increase (Fig. 5j–l). When the TPCL is pinned, the contact angle on the particle is increasing with separation until it reaches a maximum around *F*_r_ (Fig. 5m–o). The largest observed contact angles are on average *θ*_p_ = 116°, *θ*_p_ = 98° and *θ*_p_ = 78° for water, ethylene glycol and hexadecane, respectively. After the maximum contact angle is reached, the TPCL starts to move over the particle surface and *β* decreases. When the contact line is moving and the de-wetted area is decreasing, the liquid is advancing over the particle surface and the maximum contact angle should be the advancing contact angle on the particle. Indeed, the advancing contact angles observed on the particle are close to the values of advancing contact angles measured on a chemically similar flat surface (a fluorosilanized microscope glass slide) for all three liquids (water *θ*_a_ = 116°, ethylene glycol *θ*_a_ = 99° and hexadecane *θ*_a_ = 74°). Similarly, the receding contact angle on the particle surface can be observed when the liquid is receding over the particle surface. In our case this is when the capillary is formed on approach. The contact angles observed on the particle when the capillary is formed were *θ*_p_ = 87°, *θ*_p_ = 73° and *θ*_p_ = 50° for water, ethylene glycol and hexadecane, respectively. Again, these values are close to the receding contact angles measured on a similar flat surface (water *θ*_r_ = 84°, ethylene glycol *θ*_r_ = 66° and hexadecane *θ*_r_ = 51°).

On the superamphiphobic surface the contact angles are close to constant during a measurement cycle (Fig. 5p–r). The contact angles at capillary formation were *θ*_s_ = 172°, *θ*_s_ = 173° and *θ*_s_ = 164° and the largest observed angles during separation *θ*_s_ = 174°, *θ*_s_ = 175° and *θ*_s_ = 176° for water, ethylene glycol and hexadecane, respectively. Here, we see good agreement with the microscopic contact angles measured by confocal microscopy (Fig. 1d), but larger deviation from the contact angles measured by goniometry (Table 1). This again emphasizes that goniometer data are uncertain at high contact angles. The close to constant contact angles observed during force measurements is consistent with the very small contact angle hysteresis observed on macroscopic surfaces.

### Calculations of capillary-liquid–solid interactions from capillary shape and comparison with measurement

We calculate the free energy change due to capillary formation from the shape of the meniscus evaluated from confocal images. This includes contributions from the surface tension *γ*, the pressure difference across the gas–liquid interface Δ*P* and properties at the TPCL, where the contact angles determined at each separation during the dynamic force measurement, Fig. 5, are utilized. The relevant areas were also determined at each separation. A previous study^46^ concluded that the surface tension provides the major contribution. The free energy of the surface tension contribution Δ*G*_*γ*A_ under these dynamic meniscus conditions is calculated as:

1 $$\Delta {G}_{\gamma A}=\gamma \left({A}_{\mathrm{m}}+{A}_{\mathrm{p}}\mathrm{cos}{\theta }_{\mathrm{p}}+{A}_{\mathrm{s}}\mathrm{cos}{\theta }_{\mathrm{s}}\right)$$

where *A*_m_ is the capillary meniscus surface area of the gas–liquid interface, and *A*_p_ and *A*_s_ are the de-wetted areas on the particle and the superamphiphobic surface, respectively. The first term is the free energy cost of creating the gas–liquid interface, and the other two terms are the change in free energy due to de-wetting of the particle and surface, respectively.

By integrating the force-distance curve measured on approach to get the initial energy at zero distance, and then integrating the retraction force curve we can compare measurements and calculations using Eq. (1). The contact angles and surface areas were determined from confocal images (Supporting Information) and Δ*G*_*γA*_ calculated for every camera image frame during retraction.

**Figure 6:**
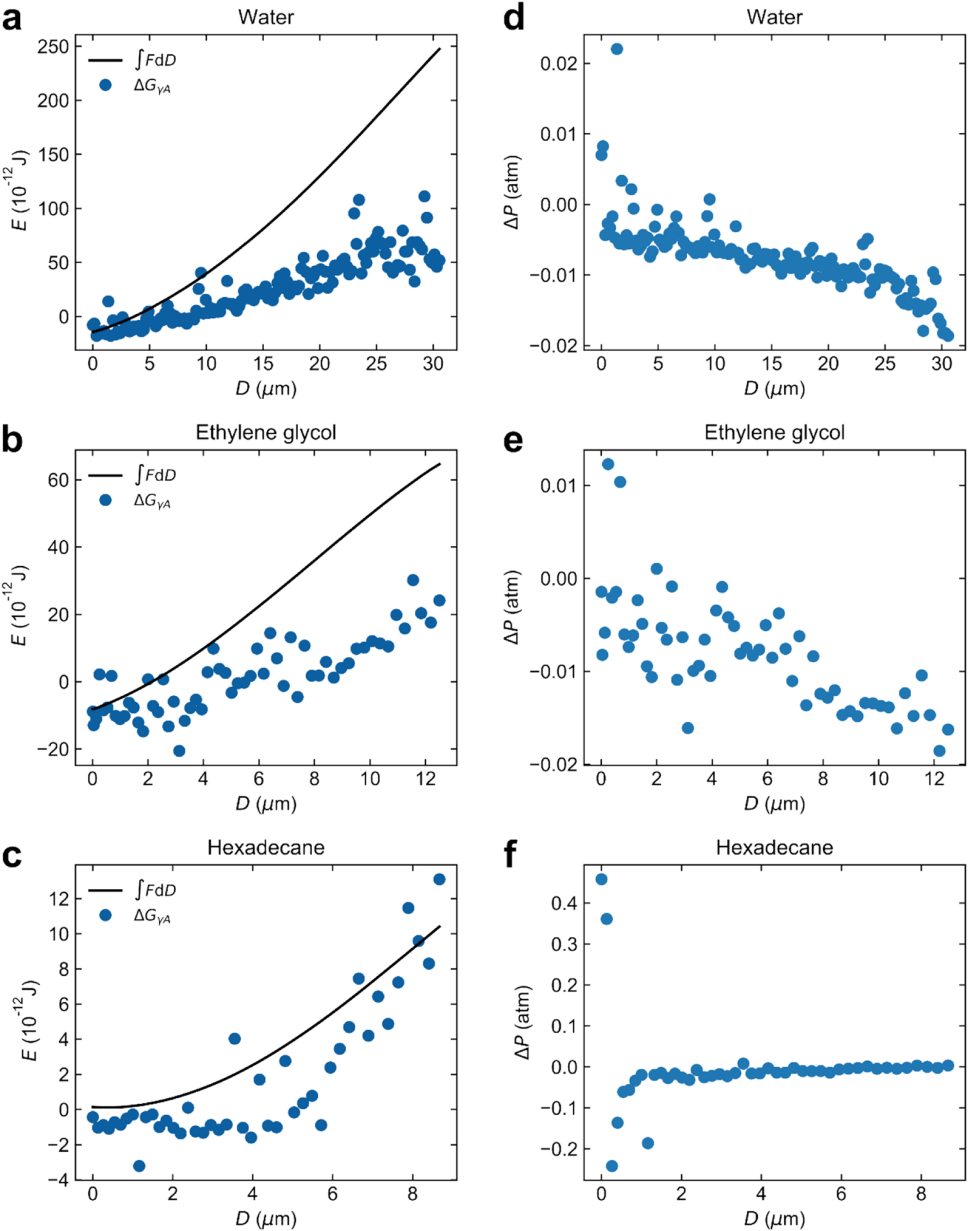
The free energy change due to gas capillary formation evaluated from the integral of the force-distance curve ∫*F*d*D* measured on retraction (solid black line) compared to the surface tension-area work Δ*G*_*γA*_ calculated using Eq. (1) (blue symbols) for measurements in (**a**) water, (**b**) ethylene glycol and (**c**) hexadecane. The piezo expansion rate was 0.2 µm s^−1^. The pressure difference across capillary gas–liquid interface Δ*P* calculated from the difference between ∫*F*d*D* and Δ*G*_*γA*_ in (**a**–**c**) as $\Delta P=-\frac{\int F\mathrm{d}D-\Delta {G}_{\mathrm{\gamma A}}}{V}$, where *V* is the capillary volume in (**d**) water, (**e**) ethylene glycol and (**f**) hexadecane.

We note that the capillary is only stable at small separations in water (*D* ≲ 4 µm, Fig. 6a) and ethylene glycol (*D* ≲ 2 µm, Fig. 6b) when the free energy is negative. When the free energy becomes positive, the gas capillary is metastable until it ruptures. The capillary can persist in a metastable state due to a high energy barrier for rupturing. In hexadecane, the capillary is metastable for the most part of the retraction (Fig. 6c).

In water and ethylene glycol we typically see a deviation between measurements and calculations at large *D* while in hexadecane, having the highest gas solubility of the three liquids, there is typically a good agreement. This suggests that gas flow from the liquid phase into the gas capillary is more important in hexadecane than in the other two liquids. The difference between the measured force and calculations may be due to contributions from the TPCLs and/or the pressure–volume work *V*Δ*P*, where *V* is the capillary volume. If the difference is caused by the *V*Δ*P* term, calculations of Δ*P* show that a very small under pressure (< 0.02 atm) in the capillary is needed to account for the difference (Fig. 6d–f). Such an under pressure would also aid the flow of gas from the gas layer on the superhydrophobic surface into the gas capillary. Considering that we report dynamic measurements, such a small under pressure is judged to be reasonable. Further experiments using different retraction speeds would be able to clarify this issue. Additionally, it is worth noting that due to error propagation, even small errors in determining capillary properties from confocal images can lead to relatively large errors in the calculated values using Eq. (1) (Fig. S8, Supporting Information).

## Summary and conclusions

The interactions between a superamphiphobic surface and a hydrophobic microparticle in three liquids with different surface tensions, water (73 mN m^−1^), ethylene glycol (48 mN m^−1^) and hexadecane (27 mN m^−1^) were elucidated. In all three liquids long-range attractive capillary forces are observed and confocal imaging revealed the formation of bridging gas capillaries. The appearance of the gas capillary coincides with the onset of the attractive force, and this occurs at larger separation for a liquid with higher surface tension due to a higher contact angle on the particle. Indeed, we observe a good consistency between receding contact angles on a chemically similar flat surface and the microscopic contact angle on the particle just after formation of the gas capillary.

On retraction, the magnitude and range of the attractive capillary forces as well as the capillary volume depend on the liquid and follow the order water > ethylene glycol > hexadecane. Free energies calculated from the size and shape of the gas capillary obtained from confocal images, as well as by integration of the measured force curves, show that the gas capillary is stable at small separation. However, the free energy becomes positive well before the gas capillary ruptures, which means that the gas capillary is metastable but persists due to a high energy barrier for rupturing.

In conclusion, we confirm for the first time that gas bridges form between interacting superamphiphobic surfaces in low-surface-tension and non-polar liquids, showing the mechanism to be physically similar as for hydrophobic surfaces interacting in water.

## Methods

### Sample preparation

Superamphiphobic coatings were prepared on high-precision thin cover glasses (No.1.5H, thickness 170 ± 5 μm, Carl Roth GmbH) using the method reported by Teisala et al.^24^.

First, a titanium dioxide–silicon dioxide nanostructured coating was applied using liquid flame spray (LFS). Hydrogen (50 L min^−1^) and oxygen (15 L min^−1^) was used as combustion gases to achieve the turbulent, high temperature flame (> 2 500 °C). A liquid feedstock of tetraethyl orthosilicate (98%, Alfa Aesar) and titanium(IV)isopropoxide (97%, Alfa Aesar) dissolved in isopropanol was injected into the flame through a custom-made spray torch at a rate of 12 mL min^−1^. The total Ti + Si concentration in the feedstock solution was 50 mg mL^−1^ with a Ti/Si weight ratio of 99/1. The coatings were applied by passing the samples 5 times through the flame spray 6 cm from the burner face with a velocity of 0.8 m s^−1^.

The nanostructured surface was then modified by chemical vapor deposition (CVD) of fluorosilane to lower the surface energy. In order to prevent degradation of the fluorosilane due to the photocatalytic activity of titanium dioxide, a thin layer of silicon dioxide was grown on the surface prior fluorosilanization. The silicon dioxide layer was applied by a gas-phase Stöber-like reaction, where the samples were placed in a desiccator together with tetraethyl orthosilicate (3 mL, 98%, Sigma-Aldrich) and ammonia (3 mL, 25%, VWR Chemicals) in two open vials at atmospheric pressure and room temperature for 4 h. After this, the samples were activated by oxygen plasma (Femto low-pressure plasma system, Diener electronic) at 300 W for 10 min. Fluorosilanization was then achieved by placing the samples in a desiccator together with 100 μL 1H,1H,2H,2H perfluorooctyl-trichlorosilane (97%, Sigma-Aldrich) and reducing the pressure to 100 mbar for 2 h. Finally, the samples were placed in a vacuum oven at 60 °C for 2 h to remove any unreacted silane.

Glass microspheres (diameter 10–40 μm, Polysciences Inc.) were glued to tipless cantilevers (NSC35/tipless/Cr-Au, Mikromasch) by a small amount of two-component glue (Epoxy Rapid, Bostik) using a micromanipulator under an optical microscope. The particles (attached to cantilevers) were fluorosilanized by CVD as described above for the superamphiphobic samples. Cantilevers were calibrated using the method described by Sader^47^ and the actual diameter of each particle was determined from SEM images.

### Surface characterization

The morphology of the nanostructured LFS-coating was imaged using scanning electron microscopy (SEM). Prior to imaging, the samples were sputter coated with a thin layer of gold to reduce surface charging. Cross-sectional images of the coating layer were recorded using a Zeiss Sigma 300 VP SEM and top-view images using a FEI Quanta 250 FEG SEM. To determine particle diameters of the colloidal probes, low-vacuum SEM (LV-SEM) images were recorded using the same FEI Quanta 250 FEG SEM operating under low vacuum (70 Pa). No surface coating was used in these cases.

X-ray photoelectron spectroscopy (XPS) analysis was performed using an AXIS Ultra^DLD^ X-ray photoelectron spectrometer (Kratos Analytical) equipped with a monochromatic Al X-ray source. The analysis area was below 1 mm^2^, with most of the signal coming from an area of about 700 × 300 μm^2^. A wide spectrum was recorded to detect elements present in the surface layer. The relative surface compositions were obtained from detailed spectra recorded for each element and quantified using atomic sensitivity factors supplied by Kratos. A high-resolution carbon C1s spectrum was curve-fitted to identify different functional groups from the chemical shifts in the carbon signal.

### Wettability

Macroscopic contact angles of purified water (Milli-Q, Type 1), ethylene glycol (99.5%, Sigma-Aldrich) and hexadecane (99%, Sigma-Aldrich) were measured using a goniometer with drop shape analysis (OCA40, Dataphysics GmbH). Roll-off angles (*RA*) were determined by measuring the tilt angle leading to roll-off for 10 μL drops. The tilt angle was increased with a rate of 0.3° s^−1^. Advancing and receding contact angles were measured by slowly (1 μL s^−1^) increasing the drop volume from 5 to 25 μL and then decreasing the volume back to < 1 μL. All contact angles were determined using the tangent fitting method in the SCA 20 software (Dataphysics GmbH) and the results of five individual measurements at different positions on the sample were averaged.

An inverted laser scanning confocal microscope (Leica TCS SP8 SMD, Leica Microsystems) with a HC PL APO CS2 40×/1.10 water objective was used to image a water droplet resting on the superamphiphobic surface.

### Laser scanning confocal microscopy combined with colloidal probe atomic force microscopy

The setup used for combined imaging and force measurements was a specially designed inverted laser scanning confocal microscope coupled with an atomic force microscope (AFM)^5,34,48^.

The confocal microscope used a 473 nm laser (Cobolt Blues™ 25 mW) and a 40 × /0.95 dry objective (Olympus). To visualize the liquid phases, fluorescent dyes were added at low concentrations. A polar dye (Atto 488, Atto-tec GmbH) was used to label water (10 mg L^−1^) and ethylene glycol (20 mg L^−1^), and a non-polar dye (N-(2,6-diisopropylphenyl)-3,4-perylene dicarboxylic acid mono imide, PMI) for labelling hexadecane (50 mg L^−1^). The fluorescence from the dyed liquid and the reflected light from the interfaces were detected simultaneously with two different detectors. The microscope was operated in *xz*-mode in which the laser was scanned along one line in the *x* direction at different heights in the *z* direction to render a 2D cross-sectional image. An average of 32 line scans was used to give the final image. Images were recorded at an acquisition rate of 1 frame s^−1^.

A JPK NanoWizard AFM (JPK Instruments AG) was used to record force-distance curves in ethylene glycol and hexadecane. During measurements the AFM head was moving with a constant piezo expansion of 0.20 μm s^−1^ during approach and retraction. For force measurements in water, the attractive forces would exceed the range of the AFM piezo scanner (about 15 μm) and full force-distance curves were recorded in a slightly different way. In these cases, an external piezo was used to move the whole AFM head towards and away from the surface (Physik Instrumente P-622.ZCL piezostage with 250 µm of closed loop operation). The cantilever bending was recorded with the AFM whilst the tip position was determined from the piezo displacement. When using the external piezo, the AFM head movement speed during approach and retraction was 0.20–0.22 μm s^−1^.

## Supplementary Information

Supplementary Information 1.

Supplementary Video 1.

Supplementary Video 2.

Supplementary Video 3.

## Data Availability

The data from the current study are available from the corresponding author upon reasonable request.
