# Supplementary material for: Effects of liquid surface tension on gas capillaries and capillary forces at superamphiphobic surfaces
Source: Sci Rep. 2023 Apr 26;13:6794. doi: 10.1038/s41598-023-33875-9 (PMC10133270; doi:10.1038/s41598-023-33875-9)
Supplement: Supplementary file 1 — Supplementary Information 1. [file 41598_2023_33875_MOESM1_ESM.docx]

Supporting Information

Gas capillaries and capillary forces at superamphiphobic surfaces: effects of liquid surface tension

Mimmi Eriksson^1,2,*^, Per M. Claesson^1,2^, Mikael Järn^1^, Viveca Wallqvist^1^, Mikko Tuominen^1^, Michael Kappl^3^, Hannu Teisala^3^, Doris Vollmer^3^, Joachim Schoelkopf^4^, Patrick A.C. Gane^5,6^, Jyrki M. Mäkelä^7^ and Agne Swerin^2,8,*^

^1^ RISE Research Institutes of Sweden, SE-11486 Stockholm, Sweden

^2^ KTH Royal Institute of Technology, School of Engineering Sciences in Chemistry, Biotechnology and Health, Department of Chemistry, Division of Surface and Corrosion Science, SE-10044 Stockholm, Sweden

^3^ Max Planck Institute for Polymer Research, Department of Physics at Interfaces, D-55128 Mainz, Germany

^4^ Omya International AG, CH-4665 Oftringen, Switzerland

^5^ Aalto University, School of Chemical Engineering, Department of Bioproducts and Biosystems, FI-00076 Aalto, Finland

^6^ University of Belgrade, Faculty of Technology and Metallurgy, Karnegijeva 4, Belgrade 11000, Serbia

^7^ Tampere University, Physics Unit, Aerosol Physics Laboratory, FI-33014 Tampere University, Finland

^8^ Karlstad University, Department of Engineering and Chemical Sciences, SE-651 88 Karlstad, Sweden

* Corresponding author: agne.swerin@kau.se

# XPS


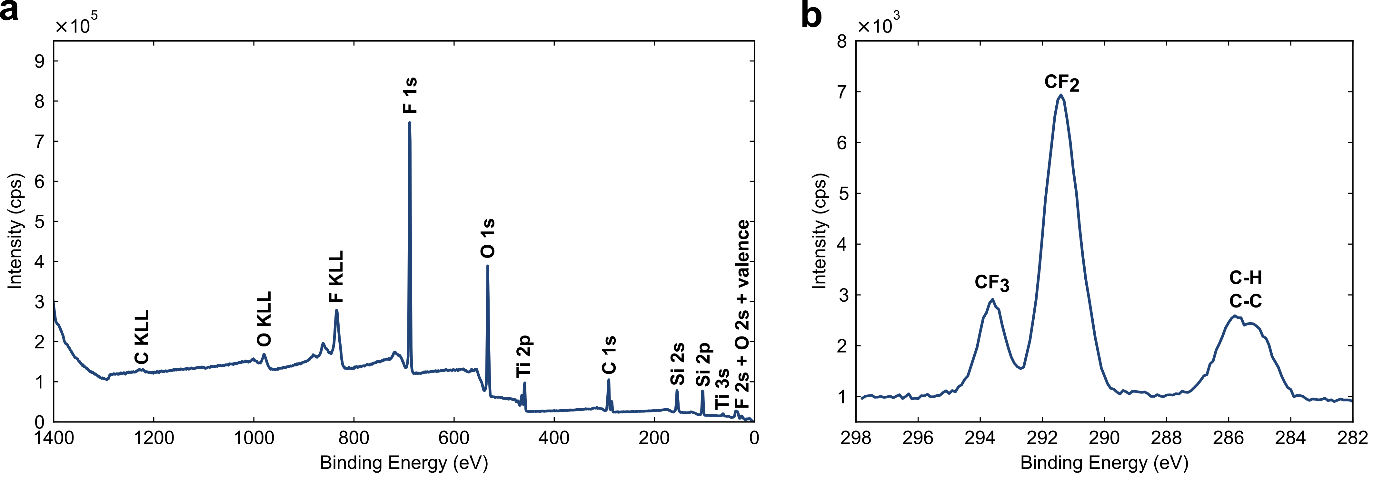


**Figure S1.** XPS spectra of the superamphiphobic sample: (a) wide spectrum and (b) high-resolution C1s spectrum.

**Table S1.** Atomic% of elements in the surface layer determined by XPS.

| Peak | Atomic % |
| --- | --- |
| F 1s | 40.6 |
| Ti 2p | 2.8 |
| O 1s | 28.2 |
| C 1s | 19.1 |
| Si 2p | 9.3 |

**Table S2.** Distribution of carbon atoms in different chemical environments as determined by XPS.

| Peak | Functional groups | Atomic % |
| --- | --- | --- |
| C1s 1-2 | C-C + C-H | 23.7 |
| C1s 3 | CF_2_ | 60.9 |
| C1s 4 | CF_3_ | 15.5 |

# Gas layer thickness under liquid droplets on the superamphiphobic surface

**
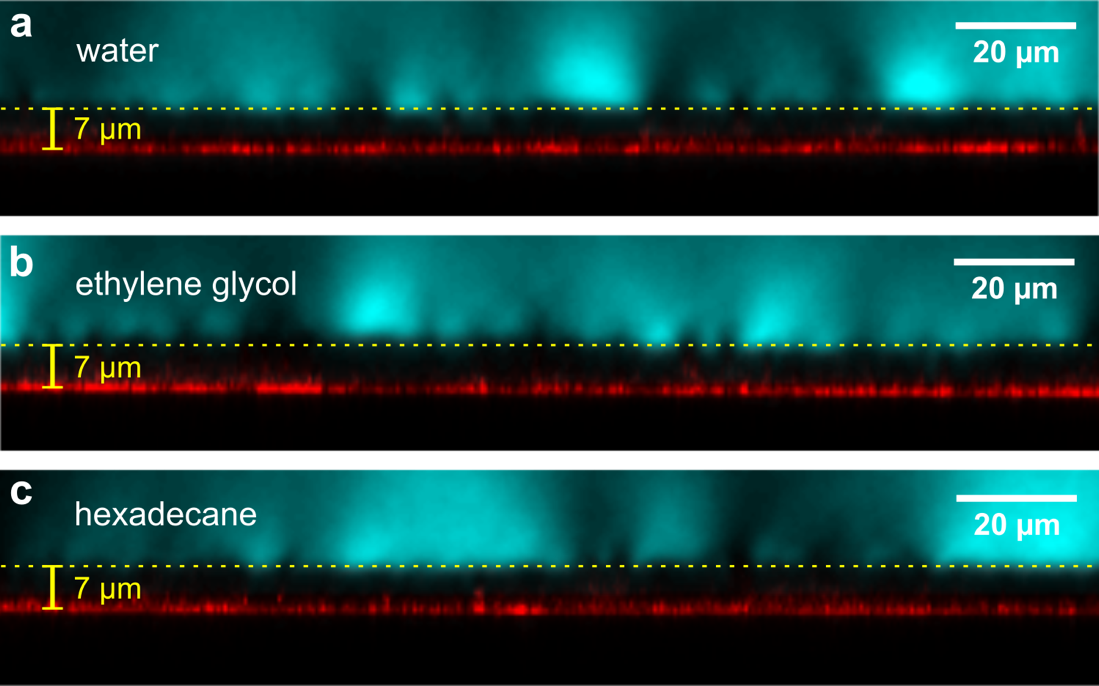
**

**Figure S2.** Laser scanning confocal microscopy images underneath fluorescently labeled drops of (a) water, (b) ethylene glycol and (c) hexadecane resting on the superamphiphobic surface. The gaseous layer is approximately 7 µm thick below all three liquids.

# Fluorescent dyes


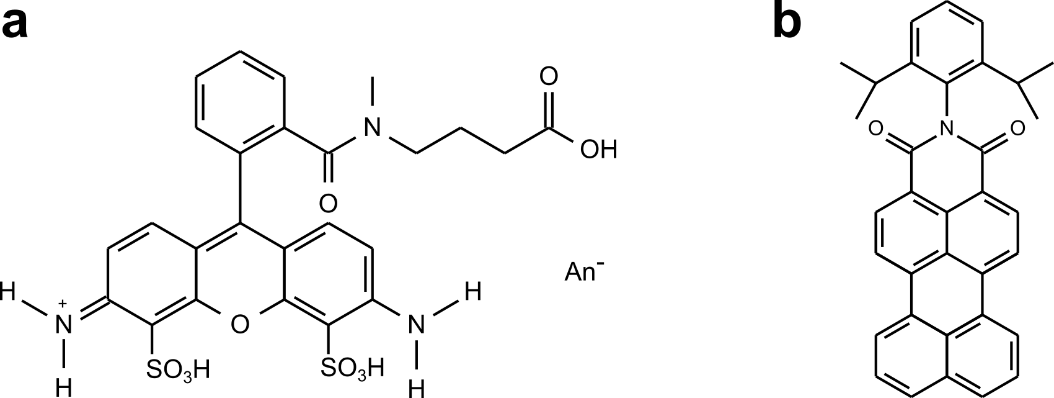


**Figure S3.** Molecular structures of (a) Atto 488 and (b) N-(2,6-diisopropylphenyl)-3,4-perylene dicarboxylic acid mono imide (PMI). Atto 488 was used for labeling water and ethylene glycol and has a maximum spectroscopic absorption wavelength at 500 nm and emission at 520 nm. PMI was used for labeling hexadecane and has a maximum spectroscopic absorption wavelength around 505 nm and emission around 525 nm [S1].

# Image analysis

Confocal images (Figure S4) were analyzed with ImageJ software. The shape of the meniscus and the liquid-gas interface was obtained from the fluorescence images. The reflection image was used to identify the position of the capillary base on the superamphiphobic surface. A circle was fitted to the position of the spherical particle.


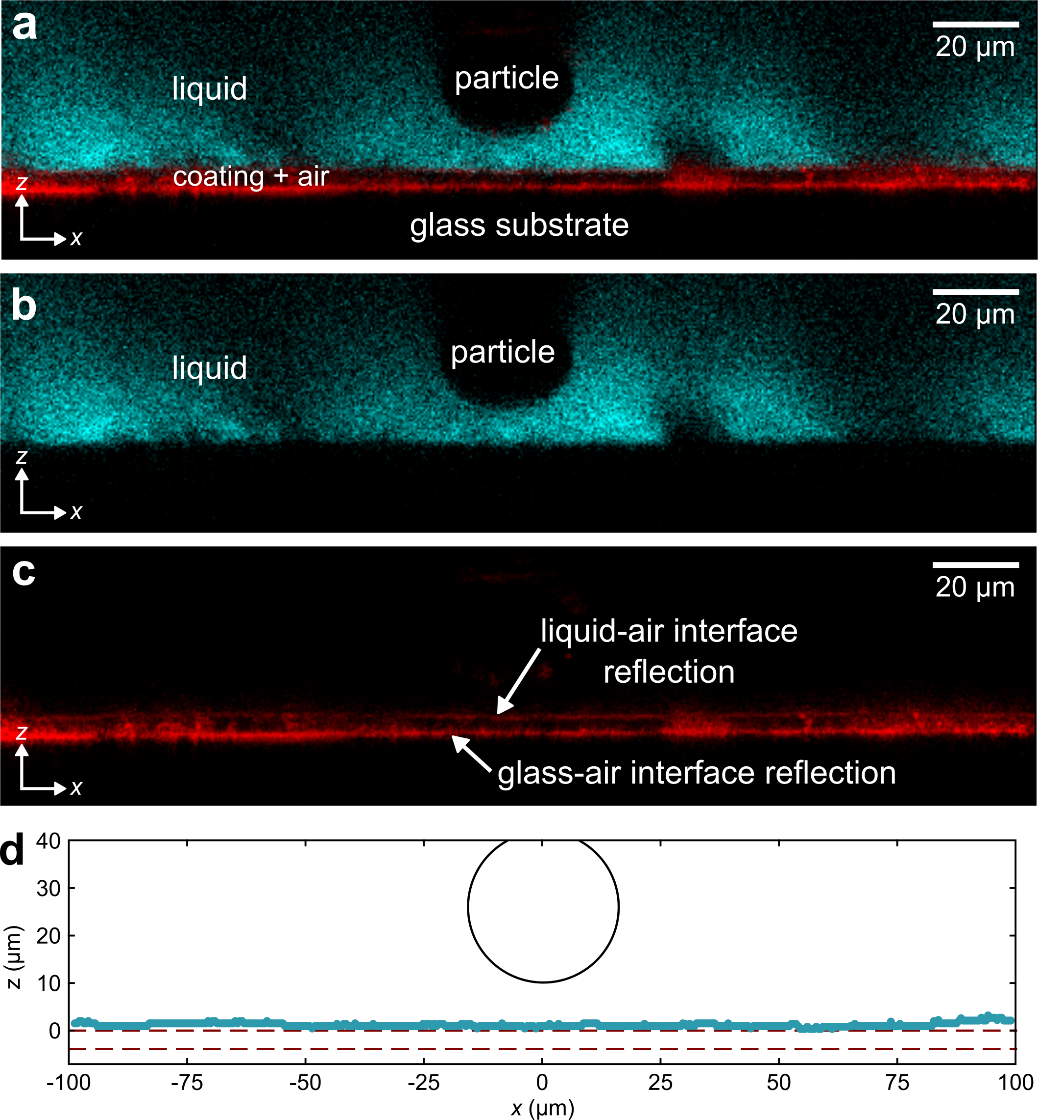


**Figure S4.** Analysis of confocal microscopy images: (a) composite image with light reflected from the interfaces in red and the liquid with fluorescent dye in cyan, (b) fluorescence image, (c) reflection image and (d) data after image analysis with the shape of the liquid-air interface (cyan symbols) and positions of the liquid-air, glass-air reflections (dashed red lines) and the particle (black circle).

# Steps in force curve on approach

In some cases, Figure S5, steps were observed in the measured forces on approach. These steps could be seen to correlate with sudden growths of the gas capillaries, and thus related to pinning/depinning of the three-phase contact line.

**
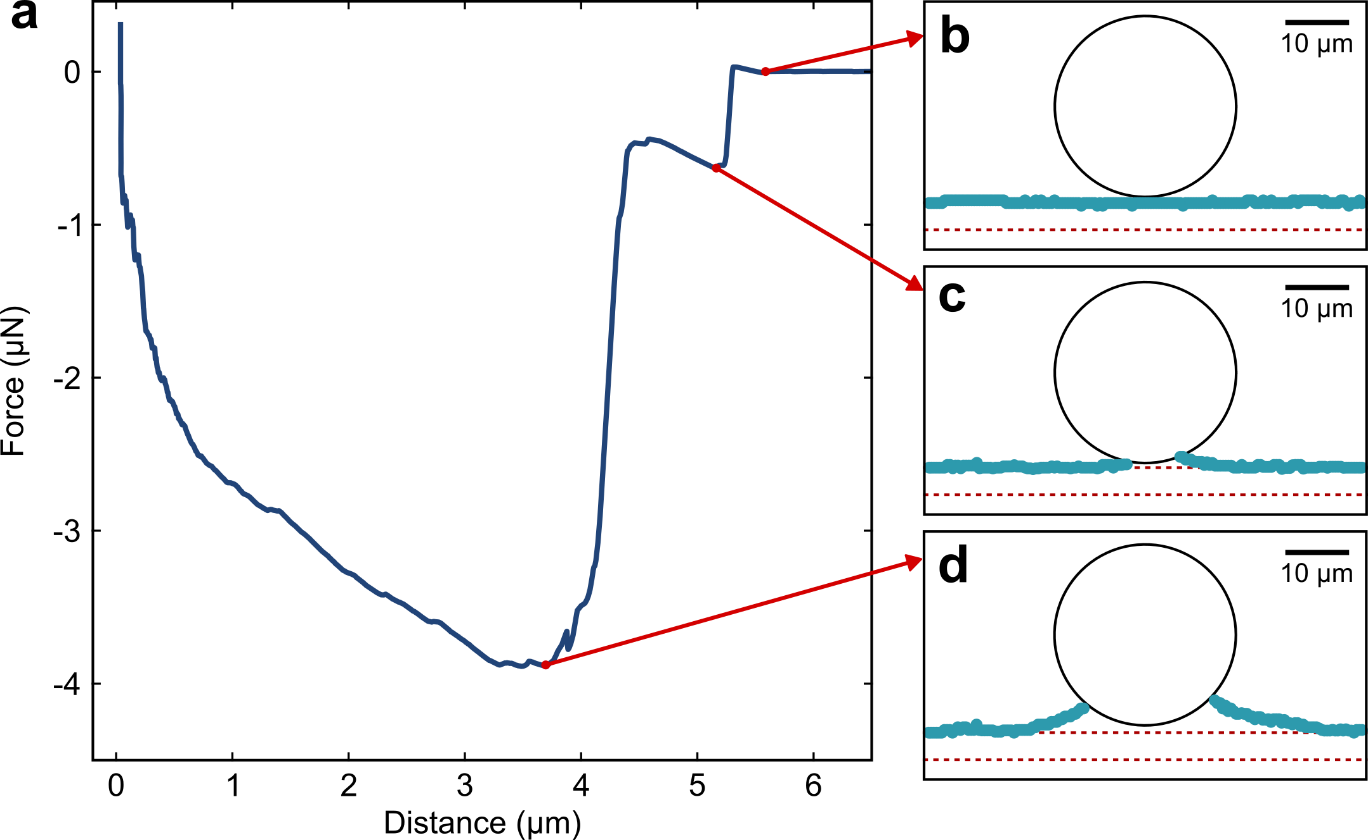
**

**Figure S5.** Example of a step in a force curve (measurement in ethylene glycol) on approach (a) with corresponding menisci shapes (b-d). A small initial capillary is formed at the first attractive step (c) and at the second step a larger capillary is formed giving rise to a stronger attractive force (d). The piezo expansion rate was 0.2 µm s^-1^.

# Weak repulsive force on approach

In hexadecane and in 50% of the cases in ethylene glycol, a weak repulsive force was observed prior to gas capillary formation, Figure S6. Image analysis allows us to identify deformation of the air layer supported by the superamphiphobic surface as the origin of this repulsion.


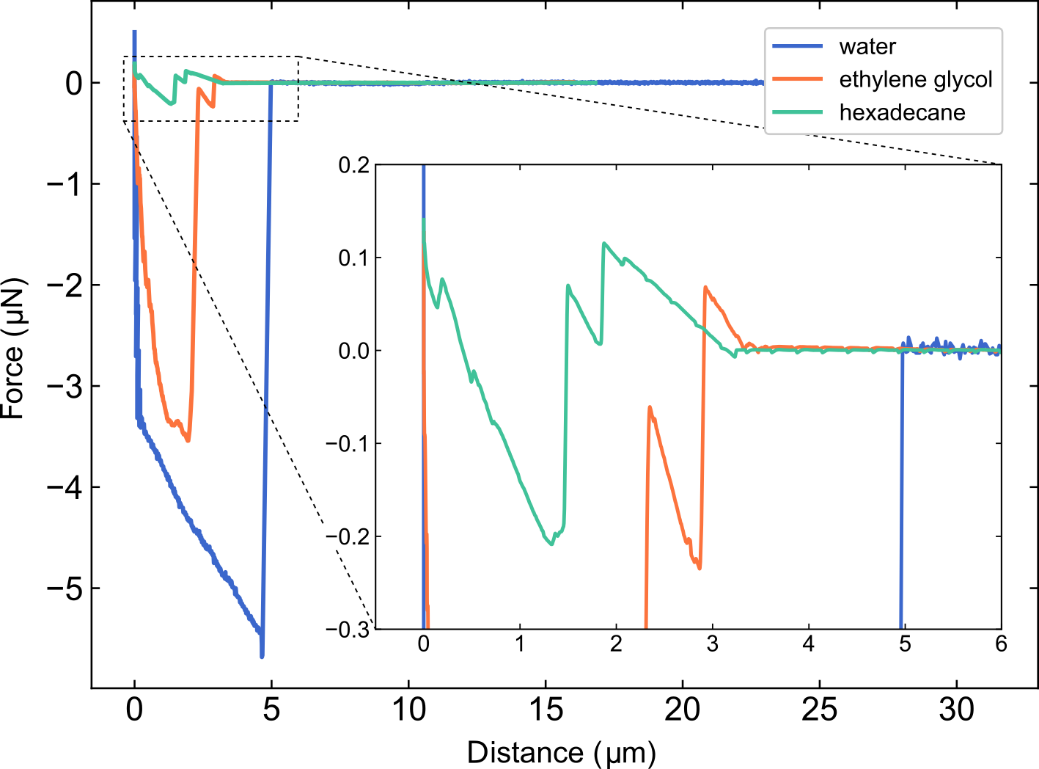


**Figure S6.** Examples of approach force curves measured in water (blue), ethylene glycol (orange) and hexadecane (green) with zoom-in on the repulsive part observed in hexadecane and ethylene glycol. Repulsion was observed in all (10 out of 10) measurements for hexadecane, in 5 out of 10 measurements for ethylene glycol and never (0 out of 10 measurements) for water. The piezo expansion rate was 0.2 µm s^-1^.

# Steps in force curve on retraction

Steps in the force curves were also observed on retraction, and just as on approach and these could in most cases be identified as being due to sudden changes in the meniscus shape, Figure S7. Thus, they are due to pinning/depinning of the three-phase contact line.


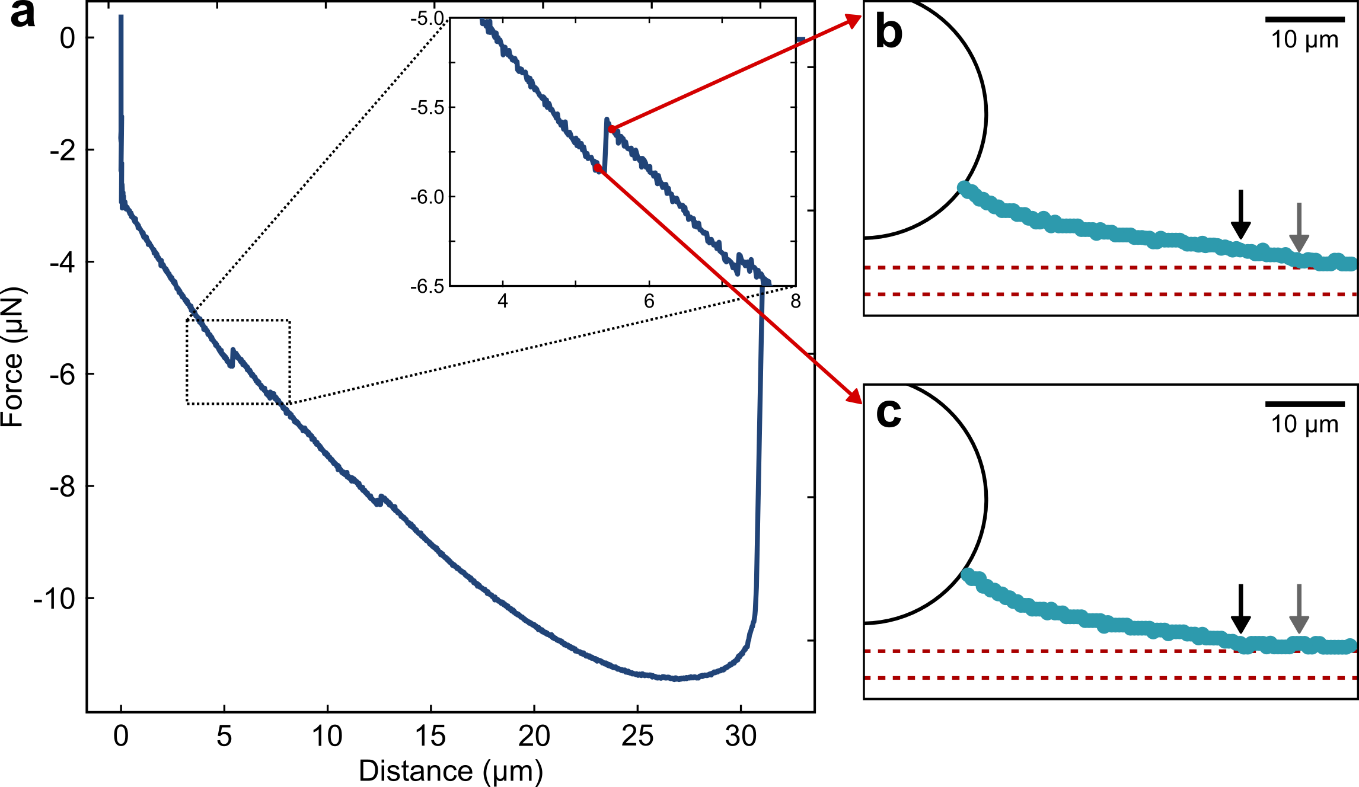


**Figure S7.** Example of steps in a force curve (measurement in water) on retraction (a) with corresponding menisci shapes (b-c). De-pinning of the three-phase contact line on the superamphiphobic surface is highlighted by the black and gray arrows in (b) and (c). The piezo expansion rate was 0.2 µm s^-1^.

## Capillary volume

To determine the gas capillary volume, *V*,we assume that the capillary is axisymmetric and that the *xz* confocal image is recorded at the center of the capillary. The volume inside the geometrical space, *V*_i_, defined by the gaseous meniscus boundaries was determined by first calculating the area of the cross sectional *xy* planes from the capillary diameter for every pixel in the *z*-direction, and then integrating the area over *z*:

$V_{i}=\int_{0}^{z} A\left( z \right) dz$ (S1)

Then, the volume occupied by the spherical cap of the particle was calculated as:

$V_{\mathrm{cap}}=\frac{\pi b\left( 3a^{2}+b^{2} \right)}{6}$ (S2)

where $b=R(1-\cos\beta)$ is the height of the cap and $a=R\sin\beta$ its radius, $\beta$ being the angle defining the de-wetted area on the particle. Finally, the capillary volume *V* was calculated by:

$V=V_{i}-V_{\mathrm{cap}}$ (S3)

## Capillary surface areas

The capillary surface area consists of three areas, the meniscus gas-liquid interface and the de-wetted areas on the particle and superamphiphobic surface, respectively. The de-wetted area on the particle, *A*_p_, was calculated from the particle radius, *R*, and the included angle defining the position of the three-phase line on the particle, *β*:

$A_{p}=2\pi R^{2}\left( 1-\cos\beta\right)$ (S4)

The de-wetted area on the superamphiphobic surface, *A*_s_, was calculated from the capillary diameter on the superamphiphobic surface, *d*_c_:

$A_{s}=\frac{\pi}{4}d_{c}^{2}$ (S5)

The area of the capillary meniscus gas-liquid interface, *A*_m_, was calculated from

$A_{m}=2\pi\int_{0}^{h} r\left( z \right)\sqrt{1+\left( \frac{dr}{dz} \right)^{2}}dz$ (S6)

where *r* is the capillary radius at each pixel in the *z*-direction and *h* is the height of the meniscus.

## Air solubility in liquids

The air solubility was calculated assuming air being equivalent to 79% N_2_ and 21% O_2_ and using Henry’s law:

$K_{H}=\frac{C}{p}$ (S7)

where *C* is the solubility, *K*_H_ Henry's constant and *p* the partial pressure of the gas.

**Table S3.** Henry’s constants for N_2_ and O_2_ in water, ethylene glycol and hexadecane at 298 K. [S2-S5]

|  | *K*_H_ (mol m^-3^ atm^-1^) | |
| --- | --- | --- |
| Liquid | N_2_ | O_2_ |
| Water [S2] | 0.66 | 1.32 |
| Ethylene glycol [S3,S4] | 0.73 | 1.29 |
| Hexadecane [S5] | 4.57 | 8.41 |

Using data in Table S3, calculations give air solubility of 0.80, 0.85 and 5.37 mol m^-3^ in water, ethylene glycol and hexadecane, respectively.

## Error analysis in calculating Δ*G*_γA_

The error in calculations of Δ*G*_γA_ using Eq. (1), and shown graphically in Figure S8, can be estimated using propagation of error analysis. The absolute error in Δ*G*_γA_ is calculated from the errors of the three terms:

$\Delta\left( \Delta G_{\gamma A} \right)=\gamma\sqrt{\left( \Delta A_{m} \right)^{2}+\left( \Delta\left( A_{s}\cos\theta_{s} \right) \right)^{2}+\left( \Delta\left( A_{p}\cos\theta_{p} \right) \right)^{2}}$ (S8)

The error for the two *A_i_*cos*θ_i_* terms (Δ(*A*_s_cos*θ*_s_) and Δ(*A*_p_cos*θ*_p_)) is calculated in the same way from the errors Δ*A_i_* and Δ(cos*θ_i_*):

$\Delta\left( A_{i}\cos\theta_{i} \right)=\left| A_{i}\cos\theta_{i} \right|\sqrt{\left( \frac{\Delta A_{i}}{A_{i}} \right)^{2}+\left( \frac{\Delta\left( \cos\theta_{i} \right)}{\cos\theta_{i}} \right)^{2}}$ (S9)

where

$\Delta\left( \cos\theta_{i} \right)=\left| \Delta\theta_{i}\sin\theta_{i} \right|$ (S10)

Next, we need to determine the errors in the surface areas. The surface area on the superamphiphobic surface, *A*_s_, is calculated from the capillary width on the surface, *d*_c_ (Eq. (S5)), and the error Δ*A*_s_ can be calculated from the error Δ*d*_c_ as:

$\Delta A_{s}=\left| \frac{\pi d_{c} \Delta d_{c}}{2} \right|$ (S11)

The surface area on the particle, *A*_p_, is calculated from the de-wetted included angle *β *Eq. (S4)), hence the error Δ*A*_p_ can be calculated from the error Δ*β * as:

$\Delta A_{p}=\left| 2\pi R^{2}\sin\beta\Delta\beta\right|$ (S12)

For the surface area of the meniscus gas-liquid interface, *A*_m_, the error is more difficult to calculate. As an estimation we assume the relative error Δ*A*_m_/*A*_m_ to be of the same order as Δ*A*_s_/*A*_s_.

If we estimate the absolute errors to Δ*d*_c_ = 1 µm and Δ*θ*_s_ = Δ*θ*_p_ = Δ*β* = 1°, calculations using Eq. (S8)-(S12) give values in the order of *G*_γA_) = 13 – 28 × 10^-12^ J in water, *G*_γA_) = 7 – 13 × 10^-12^ J in ethylene glycol and *G*_γA_) = 0.7 – 4.9 × 10^-12^ J in hexadecane. If the estimated errors instead are increased to Δ*d*_c_ = 2 µm and Δ*θ*_s_ = Δ*θ*_p_ = Δ*β* = 2°, the total errors in Δ*G*_γA_ will increase to *G*_γA_) = 26 – 57 × 10^-12^ J in water, *G*_γA_) = 13 – 26 × 10^-12^ J in ethylene glycol and *G*_γA_) = 1.4 – 9.9 × 10^-12^ J in hexadecane (Figure S9).


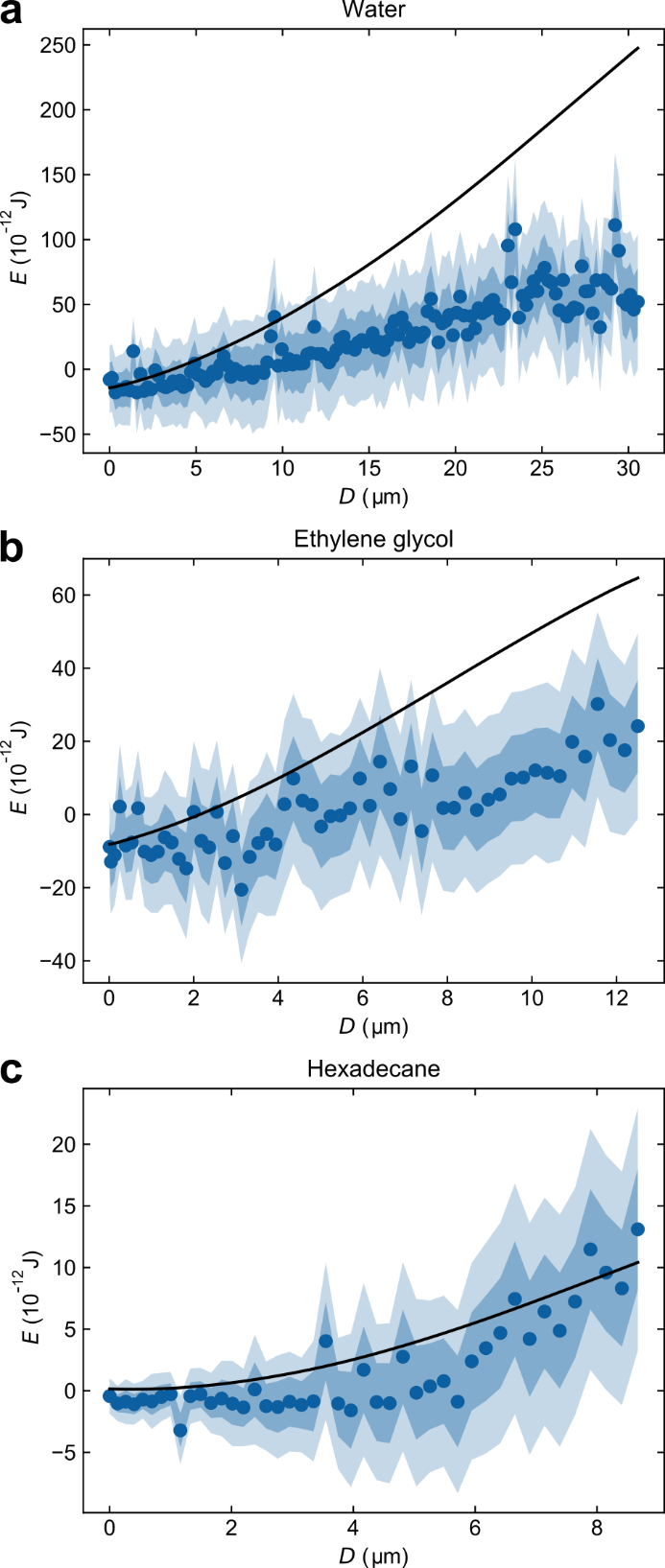


**Figure S8.** The surface tension-area work Δ*G_γA_* calculated using Eq. (1) (blue symbols) with estimation of errors for a measurement in (a) water (b) ethylene glycol and (c) hexadecane. The shaded areas represent Δ*G_γA_*+*G_γA_*) and Δ*G_γA_*-*G_γA_*), where *G_γA_*) is calculated using Eq. (S10)-(S14). The inner (darker) area is calculated from errors Δ*d*_c_ = 1 µm and Δ*θ*_s_ = Δ*θ*_p_ = Δ*β* = 1° and the outer (lighter) from Δ*d*_c_ = 2 µm and Δ*θ*_s_ = Δ*θ*_p_ = Δ*β* = 2°. The solid black lines are the integral of the force-distance curve ∫*F*d*D*. The piezo expansion rate was 0.2 µm s^-1^.

## Supporting videos

**Video S1.** Confocal images recorded during force measurement in water.

**Video S2.** Confocal images recorded during force measurement in ethylene glycol.

**Video S3.** Confocal images recorded during force measurement in hexadecane.

## Supporting references

[S1] P. Papadopoulos *et al.*, "Wetting on the microscale: Shape of a liquid drop on a microstructured surface at different length scales," *Langmuir,* vol. 28, no. 22, pp. 8392-8398, 2012.

[S2] R. Sander, "Compilation of Henry's law constants (version 4.0) for water as solvent," *Atmos. Chem. Phys.,* vol. 15, no. 8, pp. 4399-4981, 2015.

[S3] T. Sato, Y. Hamada, M. Sumikawa, S. Araki, and H. Yamamoto, "Solubility of oxygen in organic solvents and calculation of the hansen solubility parameters of oxygen," *Industrial & Engineering Chemistry Research,* vol. 53, no. 49, pp. 19331-19337, 2014.

[S4] K. Kuthan, M. Endrs̆t, and Z. Broz̆, "Mass transfer in liquid films during absorption part ii. Solubilities and diffusivities of He, N_2_ and C_3_H_8_ in aqueous ethylene glycol solutions at 25°C," *Chemical Engineering and Processing: Process Intensification,* vol. 25, no. 2, pp. 65-74, 1989.

[S5] P. J. Hesse, R. Battino, P. Scharlin, and E. Wilhelm, "Solubility of gases in liquids. 20. Solubility of He, Ne, Ar, Kr, N_2_, O_2_, CH_4_, CF_4_, and SF_6_ in *n*-alkanes *n*-C_l_H_2l+2_ (6 ≤ l ≤ 16) at 298.15 K," *J. Chem. Eng. Data,* vol. 41, no. 2, pp. 195-201, 1996.
